# Simulation the potential distribution of *Dendrolimus houi* and its hosts, *Pinus yunnanensis* and *Cryptomeria fortunei*, under climate change in China

**DOI:** 10.3389/fpls.2022.1054710

**Published:** 2022-11-14

**Authors:** Xianheng Ouyang, Haiping Lin, Shihao Bai, Jie Chen, Anliang Chen

**Affiliations:** ^1^ School of Forestry and Biotechnology, Zhejiang A&F University, Hangzhou, China; ^2^ Shanghai Center for Systems Biomedicine, Shanghai Jiao Tong University, Shanghai, China

**Keywords:** climate change, suitable distribution area, *Dendrolimus houi*, *Pinus yunnanensis*, *Cryptomeria fortunei*, MaxEnt model

## Abstract

Due to climate change, it is significant to explore the impact of rising temperatures on the distribution of *Dendrolimus houi* Lajonquiere (Lepidoptera) and its host plants, *Pinus yunnanensis* and *Cryptomeria fortunei*, and to simulate their suitable future distribution areas in order to provide a theoretical basis for the monitoring of, and early warning about, *D. houi* and the formulation of effective prevention and control policies. Based on the known distribution areas of, and relevant climate data for, *D. houi*, *P. yunnanensis*, and *C. fortunei*, their suitable habitat in China was predicted using the ENMeval data package in order to adjust the maximum entropy (MaxEnt) model parameters. The results showed that the regularization multiplier was 0.5 when the feature combination was LQHPT, with a MaxEnt model of lowest complexity and excellent prediction accuracy. The main climate variable affecting the geographical distribution of *D. houi*, *P. yunnanensis*, and *C. fortunei* is temperature, specifically including isothermality, temperature seasonality, maximum temperature of warmest month, minimum temperature of warmest month, average temperature of coldest quarter. The potential suitable distribution areas for *P. yunnanensis* and *D. houi* were similar under climate change, mainly distributed in southwest China, while *C. fortunei* was mainly distributed in southeast China. Under different future-climate scenarios, the areas suitable for the three species will increase, except for *P. yunnanensis* in the 2070s under Shared Socioeconomic Pathway 5–8.5. With climate change, all three species were found to have a tendency to migrate to higher latitudes and higher altitudes. The centroids of the areas suitable for *P. yunnanensis* and *D. houi* will migrate to the northwest and the centroids of the areas suitable for *C. fortunei* will migrate to the northeast.

## Graphical Abstract

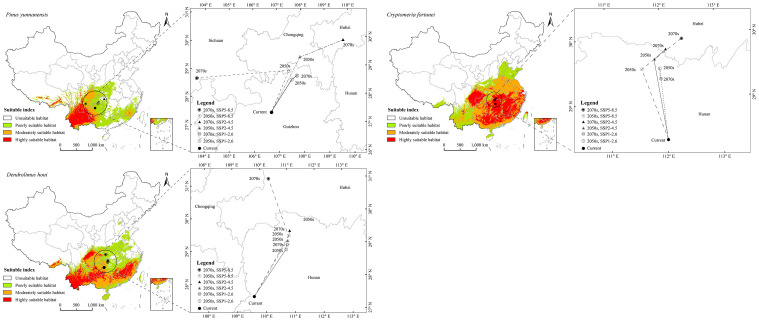

## Introduction

According to the fifth assessment report of the Intergovernmental Panel on Climate Change, the mean temperature of the Earth has risen by nearly 1°C over the last century (1906–2005) (Zhao et al., 2021), and the global climate will continue the trend of gradual warming for the rest of this century (Daba and You, 2020). The climate has the greatest influence on species distribution on Earth, and alterations of the distributions of species reflect changes in climate (Faleiro et al., 2013; Li et al., 2013). In recent years, there has been increasing attention on the impact of climate change on species distributions (Tang et al., 2021). With rising carbon dioxide levels, air temperatures are increased and natural ecosystems, including the relationship between plants and pests, are affected (Tang et al., 2021; Zhang et al., 2022). Insects-as the most species-rich and abundant organisms in the animal kingdom–have potentially important effects on agricultural production and human health, whether from the perspective of insect distributions, population densities, genetic changes, or effects on host plants and natural enemies (Porter et al., 1991; Emerson et al., 2010). Insects are variable-temperature animals, having similar internal temperatures to those of their environment. A change in environmental temperature can thus affect the distribution, behavior, development, reproduction, and survival of insects (Raza et al., 2015). Therefore, research on the response of insects to global warming has been increasingly carried out.

A species distribution model is the main method applied to evaluate the effect of a changing climate on the distribution of species (Guisan and Zimmermann, 2000). The MaxEnt (maximum entropy) model provides a prediction simulation of niche species distributions that simulates the distribution probability of species according to the distributions of those species combined with ecological parameters of the study area (Wu et al., 2021). The advantage is that, even under incomplete information on the distributions of species, the results of the method are highly accurate (Elith et al., 2011; Yi et al., 2014). At present, the MaxEnt method is commonly utilized in the distribution prediction of endangered plants (Ouyang et al., 2022) and invasive species (Yuan et al., 2021), and in the forecasting of quarantine pests (Peterson and Nakazawa, 2008), with high prediction accuracy (Kelly et al., 2014). A few studies have focused on the link between the distributions of plant diseases and insect pests and a changing climate. Models representing the distributions of species have been used to evaluate environmental factors related to an egg parasitoid and its lepidopteran hosts under climate change, and to provide a reference for formulating scientific prevention and control strategies (Wu et al., 2018). A model has been used to study the impacts of a changing climate on *Dalbulus maidis* at the global scale, with the areas suitable for *D. maidis* forecasted to decrease due to changes to the climate. The study has enabled affected regions to heighten quarantine measures and has guided the development of new varieties of *Zea mays* with increased tolerance to *D. maidis* (Santana et al., 2019). MaxEnt models have been applied to forecast the possible distribution of *Athetis lepigone*, the aim of the study being to determine the possible present distribution of the species and to calculate the risk posed by *A. lepigone* to the rest of the planet (Wang et al., 2017).

*Dendrolimus houi* Lajonquiere (Lepidoptera: Lasiocampidae) is a phytophagous pest that has wide distribution and adaptability characteristics (Liang et al., 2018). It is distributed in Yunnan, Zhejiang, Fujian, Sichuan, Guangdong, Hunan, Hubei, Guizhou, etc. in China (Yin et al., 2002). *Dendrolimus houi* harms *Pinus yunnanensis*, *Cryptomeria fortunei*, *Pinus massoniana*, *Cupressus funebris*, *Platycladus orientali*s, and other forest plants, and is a serious threat to forest ecological security and the sustainable development of forestry (Han et al., 2019). The larvae of *D. houi* eat the leaves and tender shoots of its host plants, and their food intake is considerable, each larva being able to eat 105.2 g of needles in its lifetime (Lin et al., 2017). In the case of a serious outbreak, *D. houi* will eat all the branches and leaves of its host plants and cause the host plants to die over a large area. In addition, the older larvae are large in size, with well-developed body hair, and have strong toxicity, which can cause skin allergies and even inflammation and ulceration in humans and animals (Lin et al., 2017). Because *D. houi* is increasingly harmful in forestry, several researchers have focused on biological, natural, and pest-management approaches (Han et al., 2019). However, there has been no previous assessment of the impacts of a changing climate based on simulating different scenarios of climate change and considering host plants. *P. yunnanensis* and *C. fortune* are the main host plants. *D. houi* is frequently found in Yunnan, Zhejiang, Fujian and other places, seriously endangering the key ecological areas, especially the global climate change nowadays (Kong et al., 2007; Hua et al., 2019). *Pinus yunnanensis* is the dominant native tree species in Yunnan, China, and is a significant part of Yunnan landscapes (Tang et al., 2013). *Cryptomeria fortunei* is an economically important coniferous species distributed among the different regions south of the Yangtze River (Wang et al., 2021).

The present study used *D. houi* and its host plants, *P. yunnanensis* and *C. fortunei*, as research subjects in order to explore their current suitable distributions and the dominant climate variables involved in that, and to estimate alterations in their distributions under scenarios of future changing climate, so as to provide a basis for dealing with the effects of a changing climate on *D. houi*, and subsequently its control.

## Methods and materials

### Species occurrence data

**Figure 1:**
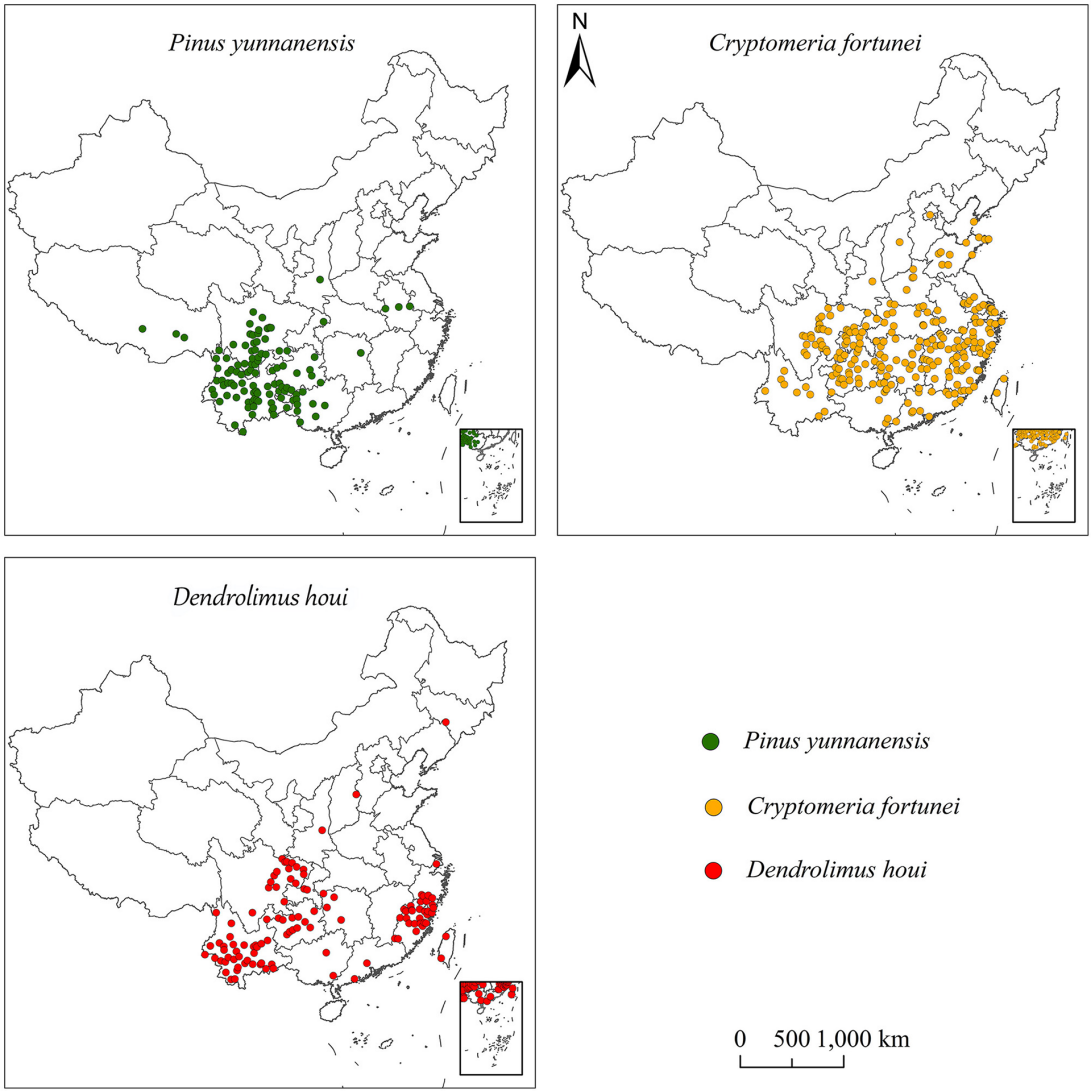
Occurrence data for *P. yunnanensis*, *C. fortunei*, and *D. houi* in China.

The data for the occurrence of *D. houi* were derived from published literature. The occurrence records for *C. fortunei* and *P. yunnanensis* were sourced from the Global Biodiversity Information Facility, the Chinese Virtual Herbarium, and the China National Specimen Information Infrastructure (available at http://www.gbif.org; http://www.cvh.ac.cn; http://www.nsii.org.cn). Data without detailed geographical location and duplicate specimen information were deleted. Google Earth (http://ditu.google.cn/) was utilized to source the latitude and longitude of the distribution points. Ultimately, a total of 112,238,111 occurrence datapoints were obtained for *P. yunnanensis*, *C. fortunei*, and *D. houi* in order to establish the models (**Figure 1**).

### Environmental parameters

Climate parameters (bio1–bio19) were downloaded from the WorldClim dataset v.2.1 (http://www.worldclim.org/) with a 2.5'resolution for the current and future period scenarios (1970–2000 and 2050s, 2070s) (Fick and Hijmans, 2017) (**Table S1**). Bioclimatic data in the 2050s represented averages from 2041 to 2060, whereas that in the 2070s represented averages from 2061 to 2080. The future-climate model chosen in the present study was the Coupled Model Intercomparison Project Phase 6’s (CMIP6). Beijing Climate Center Climate System Model (BCC-CSM2-MR) developed by the National Climate Center is used to predict the distributions of the three species in China. This model has been widely applied in East Asia (Xin et al., 2013; Tang et al., 2022). Analyses of the representative concentration pathways and the shared socioeconomic pathways (SSPs) were conducted using the ScenarioMIP (CMIP6-scenario Model Intercomparison Project), resulting in new forecast scenarios that are likely closer to reality (Eyring et al., 2016). The present study used the SSP1–2.6, SSP2–4.5, and SSP5–8.5 scenarios as the future-climate scenarios in this study. The SSP1–2.6 scenario uses a path of sustainable development characterized by low concentrations of greenhouse gases, and smaller usage of matter, resources, and energy. SSP2–4.5 assumes the continual development of the current socioeconomic model with medium emissions of greenhouse gasses. Finally, while the SSP5–8.5 scenario assumes a fully-developed economy, energy-intensive activities remain due to economic drivers and greenhouse gas emissions remain high (Eyring et al., 2016; Riahi et al., 2017). Due to the direct correlation of environmental variables, and in order to avoid overfitting of the MaxEnt model, Spearman correlation analysis was carried out in ArcGIS 10.2.2 for all environmental variables (Yang et al., 2013). A correlation coefficient between two environmental parameters ≥ 0.8 resulted in environmental variables with small rates of contribution being discarded, and the respective six, six, and seven environmental factors associated with *D. houi*, *P. yunnanensis*, and *C. fortunei* that were ultimately selected were used for analysis in the MaxEnt model (**Table 1**).

**Table 1:** Major bioclimatic variables affecting the habitat distribution of *Dendrolimus houi* and its hosts, *Pinus yunnanensis* and *Cryptomeria fortunei*, in China.

Species	Environmental variables
*Dendrolimus houi*	Bio6, bio4, bio5, bio3, bio12, bio15
*Pinus yunnanensis*	Bio4, bio11, bio3, bio12, bio5, bio15
*Cryptomeria fortunei*	Bio6, bio5, bio3, bio16, bio14, bio15, bio7

### Model optimization

We used MaxEnt V3.4.1 to simulate the possible geographical distributions of species under different periods. The number of repetitions was set to 10. The method used for extracting test samples was cross-validation–a method that divides all modern records of species into 10 subsets, one of which being a validation set, while the other nine are used as training sets, which improves the utilization of the data.

The MaxEnt model was optimized using the ENMeval packet in R v.3.61 (Muscarella et al., 2014). The parameter of regulation multiplication (RM) was set to 0.5–4, with a 0.5 interval each time. There are a total of eight RM parameters. For feature combination (FC), the MaxEnt model provided six features–LQ, L, LQH, H, LQHPT, and LQHP, where L = linear, H = hinge, P = product, Q = quadratic, and T = threshold (Phillips and Dudík, 2008). The ENMeval package was utilized to assess the combined 48 parameters, and finally evaluated the fitting and complexity by using the delta corrected Akaike information criterion (AICc) model. A lowest AICc (delta AICc = 0) represented the best model parameter combination; this was then used for MaxEnt modeling (Warren and Seifert, 2011). MaxEnt V3.4.1 software was utilized to forecast and study suitable habitats for *D. houi*, *P. yunnanensis*, and *C. fortunei*, with 75% and 25% of the points selected for model training and model verification, respectively. The Jackknife option was selected in the setting for environmental parameters. The output of the analysis was in logistic format and ASCII-type files. After the optimization was completed, the optimization parameters were used for simulation and prediction. Model accuracy was assessed using the area under the receiver operating characteristic curve (AUC), under which its value was proportional to the model prediction accuracy. The AUC ranged between 0 and 1 and was proportional to the accuracy of the model (Hanley and McNeil, 1982). In general, AUC values between 0.5– 0.6, 0.6–0.7, 0.7–0.8, 0.8–0.9, and 0.9–1.0 represent unacceptable, poor, fair, good, and excellent model accuracy, respectively (Zhao et al., 2021).

We used ArcGIS 10.2.2 software to carry out suitability division and visualization of the simulated distribution areas. Based on the threshold generated by the MaxEnt software, the habitat suitability index was divided. The potential habitats were placed into four categories: 0.5–1, representing highly suitable habitat; 0.3–0.5, representing moderately suitable habitat; 0.1–0.3, representing poorly suitable habitat, and; and 0–0.1, representing unsuitable habitat (Yan et al., 2021).

### Multivariate environmental similarity surface analysis

The MESS calculates how similar points around a predictor set relate to a reference point set. If negative values appear, this indicates that the value of a minimum of one variable is higher than the environment of the reference point set in a particular time range, referred to as a “climate anomaly”. A value of this of 100 represents a climate environment that is wholly consistent with the reference layer. Multivariate similarity is represented by its lowest similarity relative to each variable, including the variable with the least similarity, representing the variable with the highest anomaly, which may be an important factor regulating species migration (Elith et al., 2010). This step used the density tool, Novel, in the MaxEnt model.

## Results

### Optimization of the model and its accuracy

**Figure 2:**
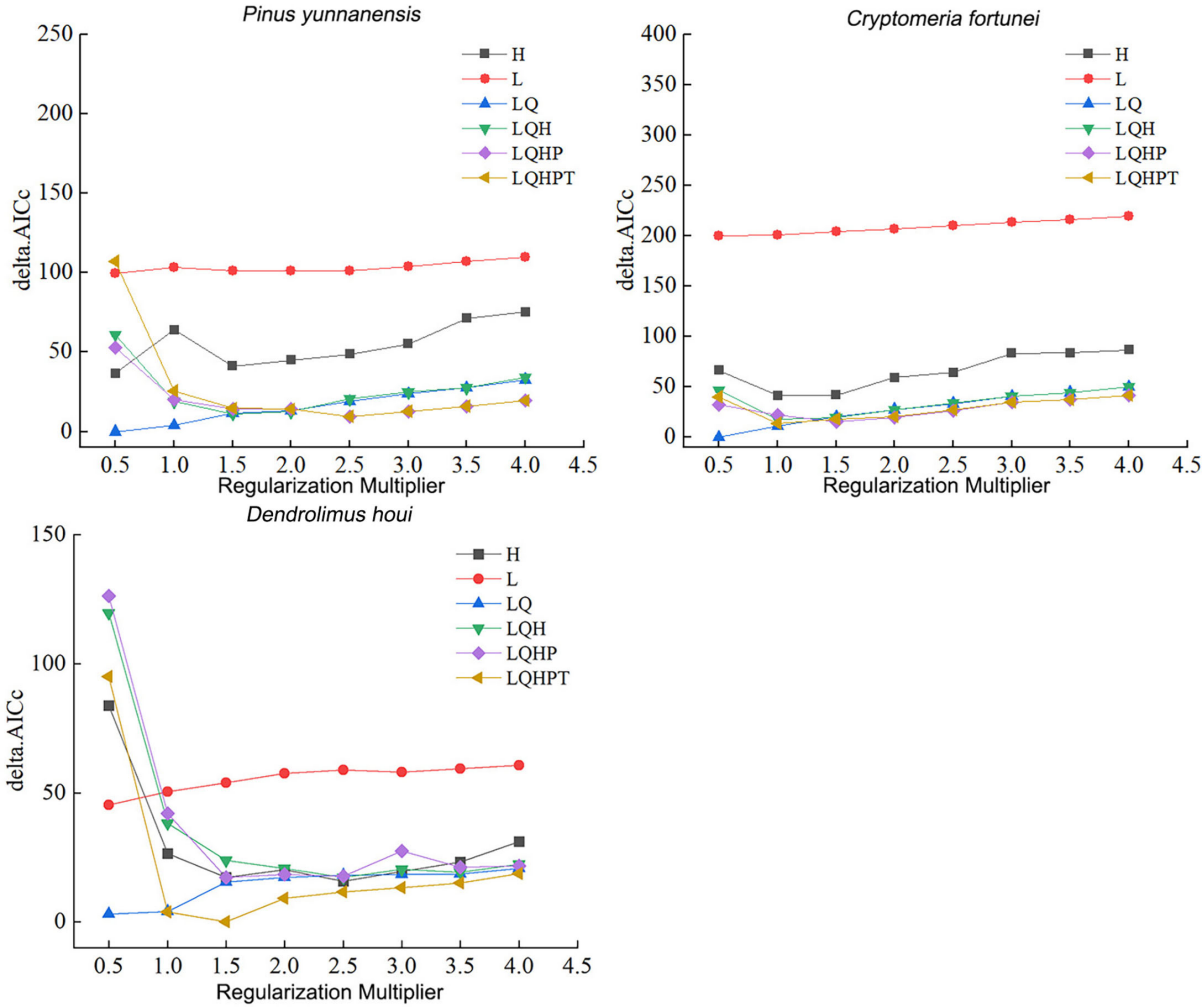
Delta AICc for *P. yunnanensis*, *C. fortunei*, and *D. houi* from models under different parameter combinations.

The RM and FC parameters in the MaxEnt model were set to default values of 1 and LQHPT, respectively. ENMeval was used to optimize the parameters in MaxEnt. As shown in **Figure 2**, the minimum AICc was obtained under RM and FC values of 0.5 and LQ, respectively, i.e. delta AICc = 0. The AIC indicated that the lowest model complexity was under this parameter. This indicated that this model showed the lowest overfitting. Therefore, the present study chose RM and FC values of 0.5 and LQ, respectively as optimal model parameters.

The MaxEnt model under the above parameter setting was used to forecast potential suitable habitat for *P. yunnanensis*, *C. fortunei*, and *D. houi*. The AUC of the *P. yunnanensis*, *C. fortunei*, and *D. houi* training data were 0.954, 0.911, and 0.945, respectively (**Figure S1**), indicating accuracy of the model for all three species.

### Assessment of important climatic parameters

The percentage contribution, which is a way of showing the relative contribution of each variable to the forecasts, is utilized to separate the impacts of the various variables on the possible distributions (**Table 2**). In the model for *D. houi*, temperature seasonality (bio4), maximum temperature of warmest month(bio5), and minimum temperature of warmest month (bio6) had the maximum contribution, with a total contribution of 93.8%. Meanwhile, isothermality, seasonality of temperature, and average temperature of coldest quarter (bio3, bio4, and bio11, respectively) were the three key variables for forecasting the likely distribution of *P. yunnanensis*, with a total contribution exceeding 93.2%. The minimum temperature of the warmest month, maximum temperature of warmest month, and isothermality (bio6, bio5, and bio3, respectively) were the key variables for forecasting the likely distribution of *C. fortunei*, with a total contribution exceeding 95.9%.

**Table 2:** Climatic variables selected by the MaxEnt model.

Bioclimatic variable (symbol)	Contribution (%)		
	*Pinus yunnanensis*	*Cryptomeria fortunei*	*Dendrolimus houi*
Isothermality (bio2/bio7) (×100) (bio3)	12.6	3.3	4.2
Temperature seasonality (standard deviation ×100) (bio4)	44.5	–	12.1
maximum temperature of warmest month (bio5)	2.1	3.5	4.7
Minimum temperature of warmest month (bio6)	–	89.1	77
Annual mean temperature range (bio7)	–	0.2	–
Average temperature of coldest quarter (bio11)	36.1	–	–
Annual precipitation (bio12)	3.6	–	1.7
Precipitation of driest month (bio14)	–	0.6	–
Precipitation seasonality (coefficient of variation) (bio15)	1	0.6	0.3
Precipitation of wettest quarter (bio16)	–	2.5	–

### Current potential distribution

**Figure 3:**
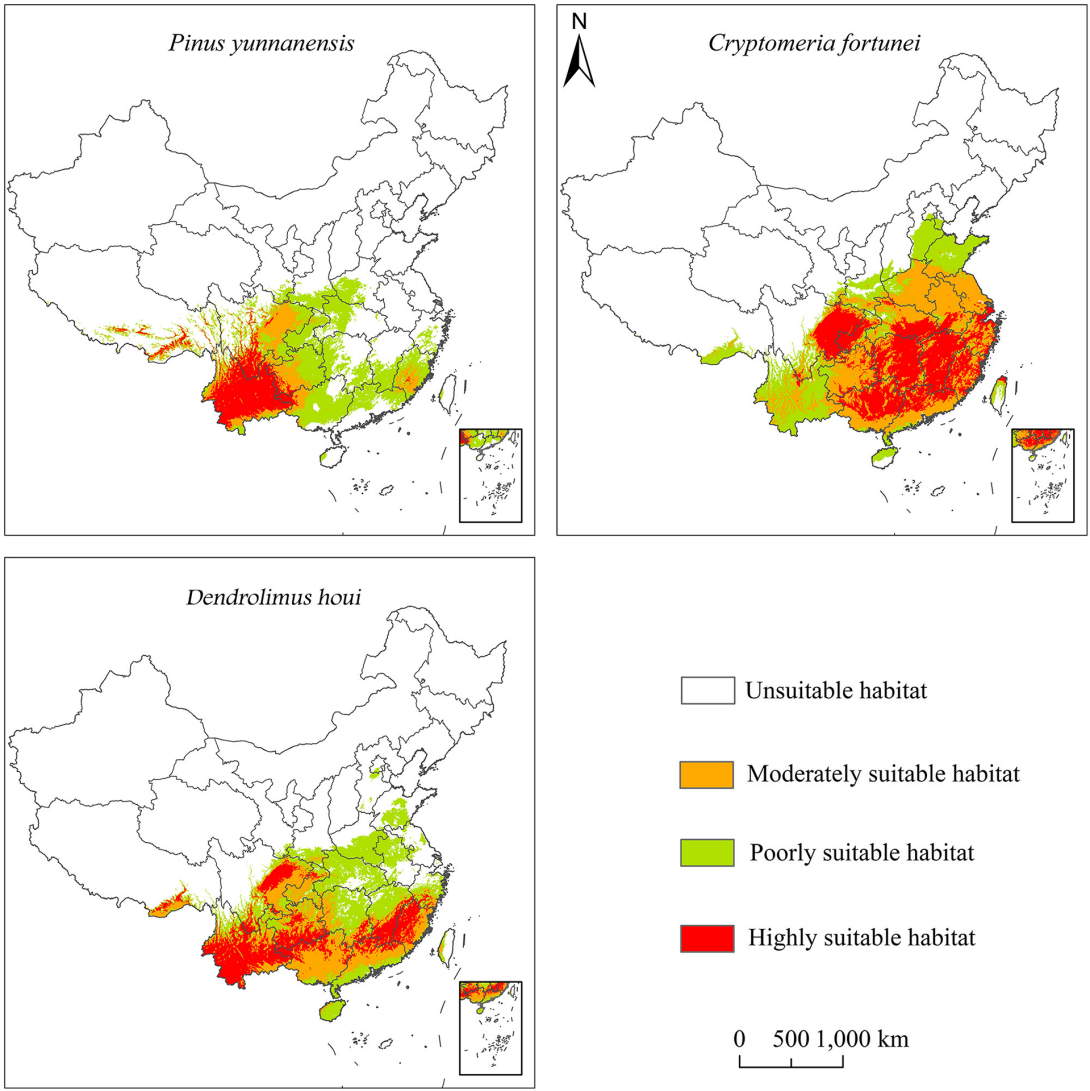
Potential habitats suitable for *P. yunnanensis*, *C. fortunei*, and *D. houi* in China.

The biggest intersection with the spatial distribution of *P. yunnanensis* was shown by *Dendrolimus houi*. Suitable habitats (high to low suitability) for both *D. houi* and *P. yunnanensis* concurrently were mainly distributed in southwest China (**Figure 3**). The provinces containing the distribution points were, in order of more to fewer, Yunnan, Guizhou, Sichuan, Guangxi, Zhejiang, Fujian, Guangdong, Hunan, and Hubei. The distribution range for *C. fortunei* was mainly in southeast China. The provinces containing the distribution points were, in order of more to fewer, Zhejiang. Fujian, Anhui, Jiangxi, Guizhou, Hunan, and Shaanxi.

### Future changes in suitable habitats

**Figure 4:**
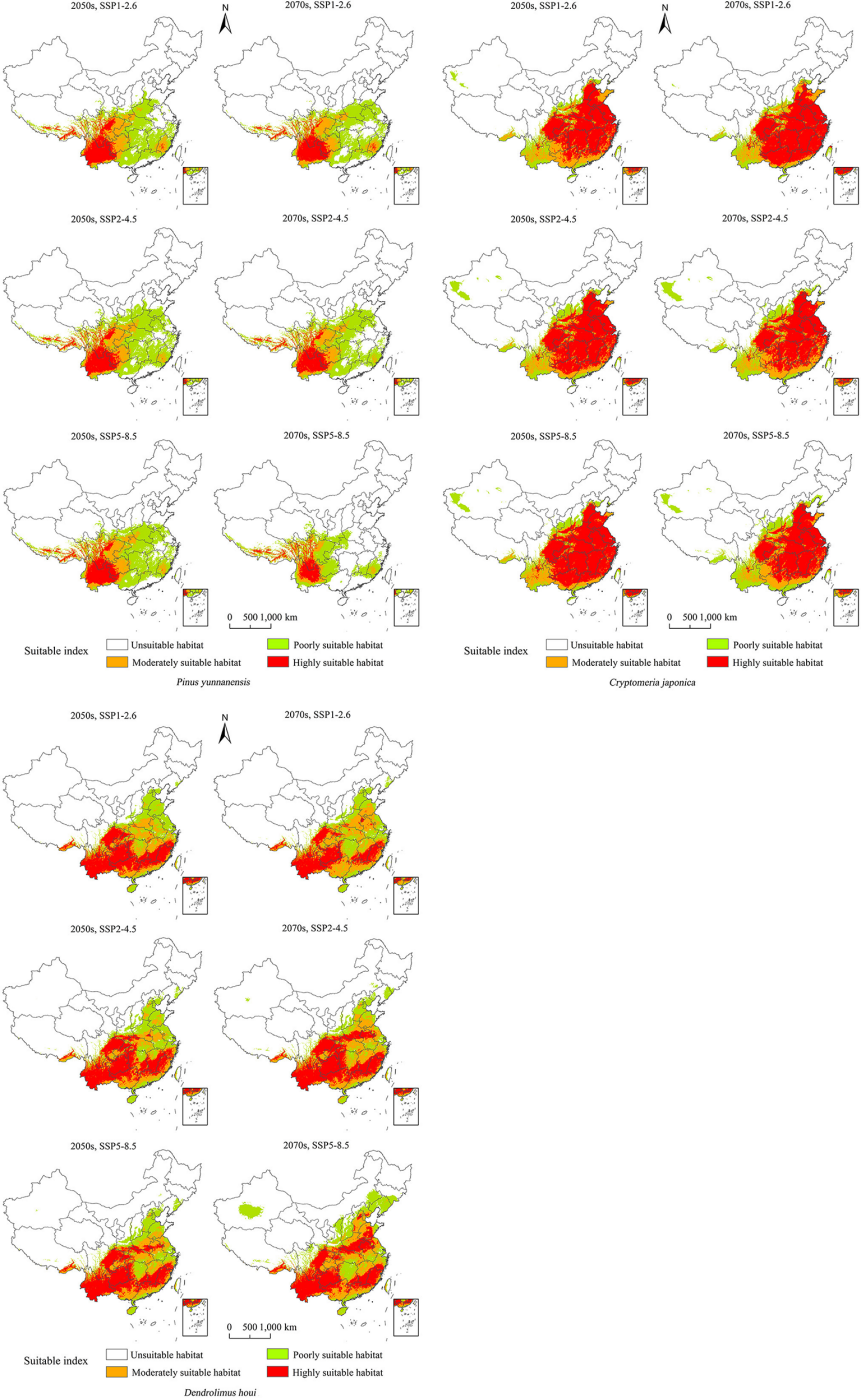
Potentially suitable climatic distributions for *P. yunnanensis*, *C. fortunei*, and *D. houi* under different climate-change scenarios.

The forecasts under different future climate scenarios showed increases in the predicted total area suitable for the three species in the 2050s and 2070s to different degrees compared with the current period, except for *P. yunnanensis* under SSP5–8.5 in the 2070s (**Figure 4**). A changing climate will result in increased or reduced risks of outbreaks of forest diseases by influencing species distributions (**Table S2**). There will be a continuous increase in the area of distribution of *D. houi* under a changing climate, with the hazard area being more extensive. Meanwhile, *P. yunnanensis* and *C. fortunei* will expand northwestwards by 2050, with their area of distribution continuing to increase. The areas of distribution of *P. yunnanensis* and *C. fortunei* will show a declining trend after 2050. *Dendrolimus houi* will expand northeastwards in the future, as will *P. yunnanensis*.

**Figure 5:**
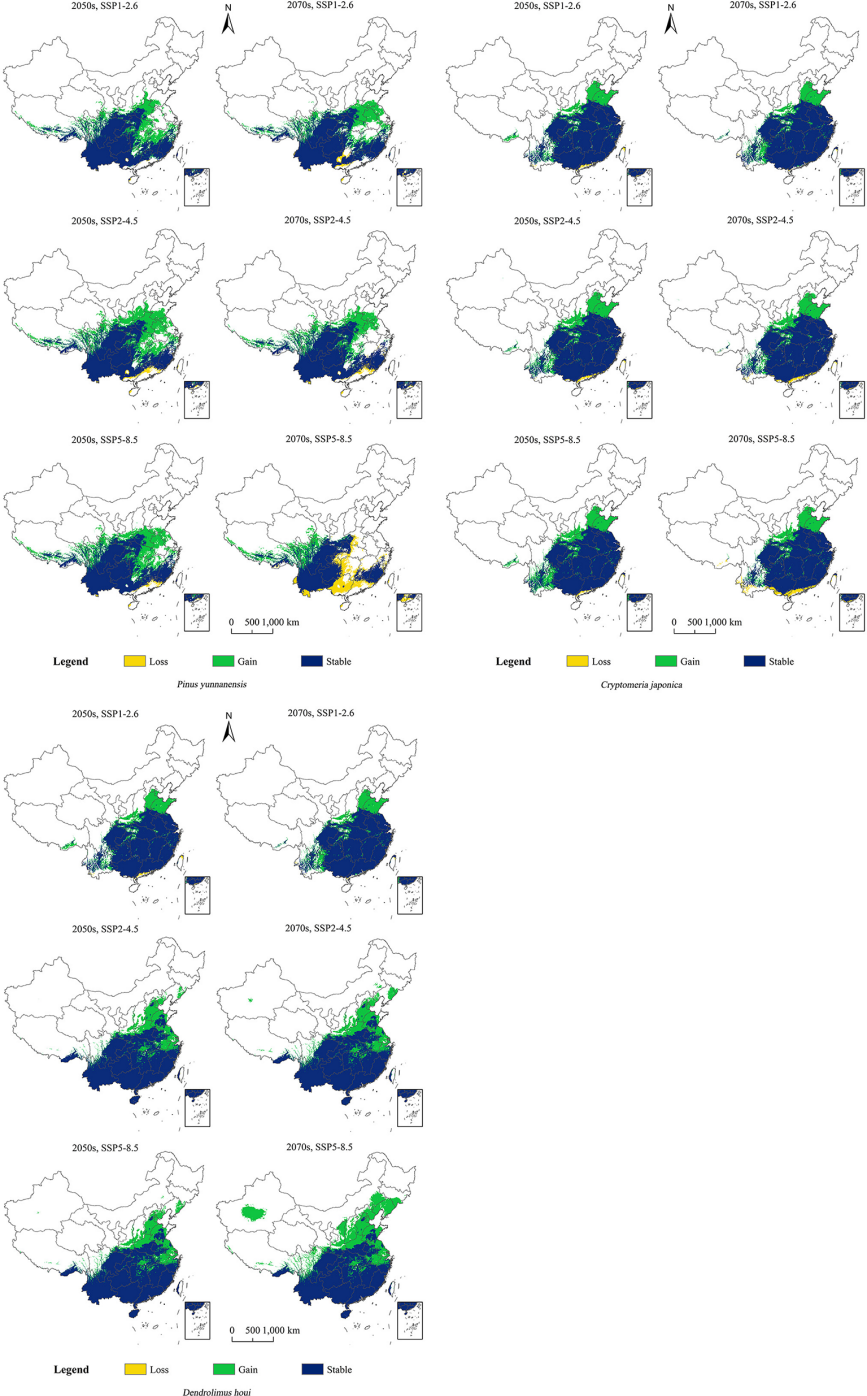
Changes in the potential geographical distributions of *P. yunnanensis*, *C. fortunei*, and *D. houi* under different climate-change scenarios.

It can be seen from **Figure 5** that the distribution of *D. houi* will increase in Shandong, Hebei, and Jiangsu to varying degrees under the various scenarios of future changing climate. The increase in suitable habitat area for *D. houi* in Yunnan in the 2070s under SSP5–8.5 will be the greatest, with an area of 138.40×10^4^ m^2^ (**Table S3**). Even if the habitat suitable for *D. houi* will be lost to some extent in the future, the area of the increase is greater than the area of the loss. Under the SSP5–8.5 future climate-change scenario, the loss of suitable habitat area for *P. yunnanensis* will be greater than the area gained in the 2070s, with the areas of loss being located in Guangxi, Guangdong, and part of Yunnan. Under the future-climate scenario, the increase in habitat area suitable for *C. fortunei* will be greater, and mainly located in Shandong and Hebei. Under SSP5–8.5 in the 2050s, the increase in habitat area suitable for *D. houi* will be the greatest, with an area of 62.06×10^4^ m^2^.

**Figure 6:**
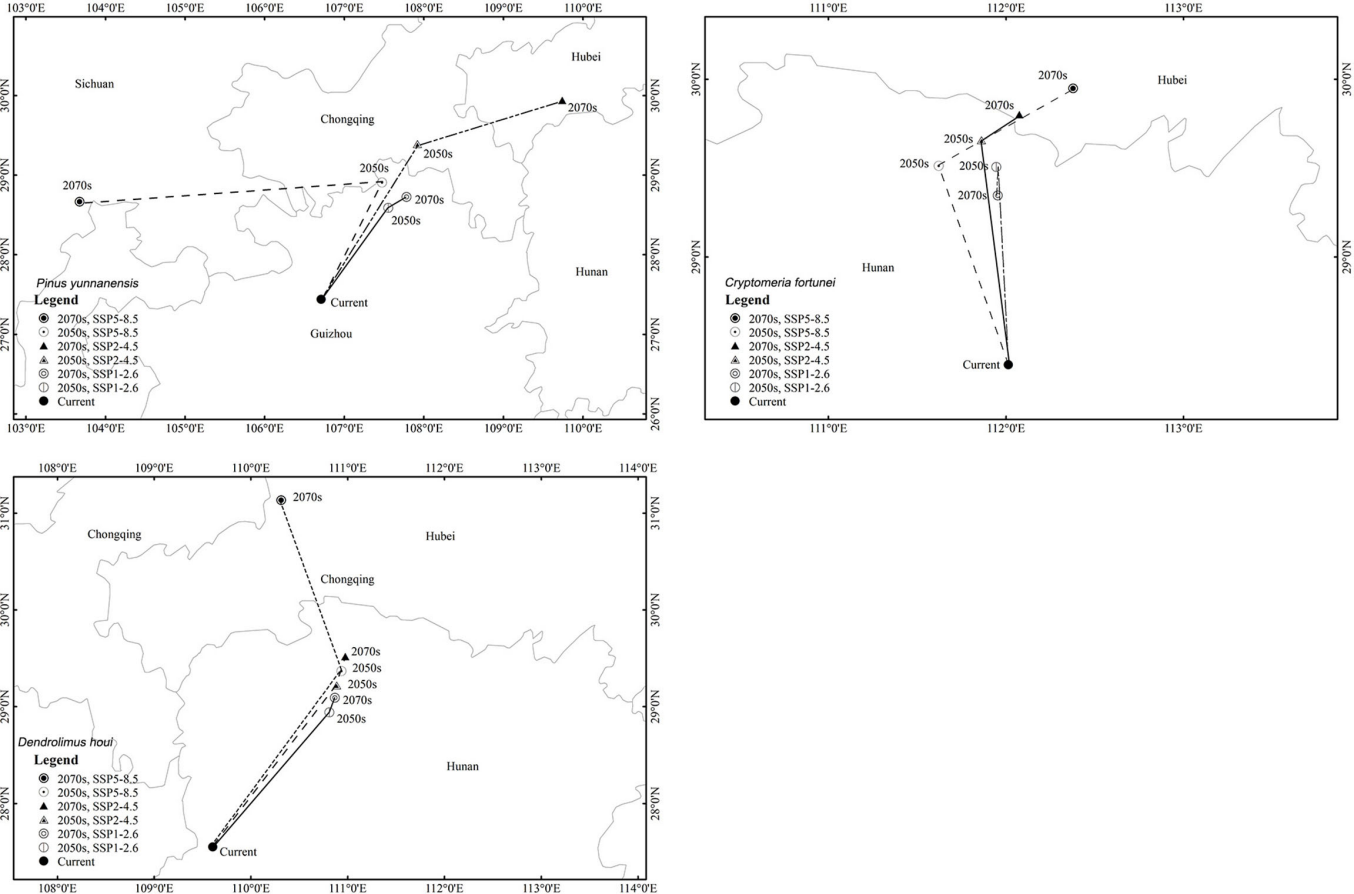
Spatial changes in the geometric centroids of suitable habitat areas by 2050 and 2070 under two different scenarios of a changing climate.

The suitable-distribution ranges for *P. yunnanensis* and *D. houi* were similar in latitude and longitude under the three scenarios of future climate change (**Figure 6**; **Table S4**). In the 2050s, under SSP2–4.5, the 2070s under SSP2–4.5, and the 2070s under SSP5–8.5, the latitudinal and longitudinal migration was relatively obvious. All three species had a tendency to spread to higher-latitude and higher-altitude areas. The suitable-distribution range for *P. yunnanensis* and *D. houi* mainly shifted to the northeast. However, the suitable-distribution range for *C. fortunei* mainly shifted to the northwest.

### Assessment of the multivariate environmental similarity surfaces of areas of potential distribution under future changes in climate

**Figure 7:**
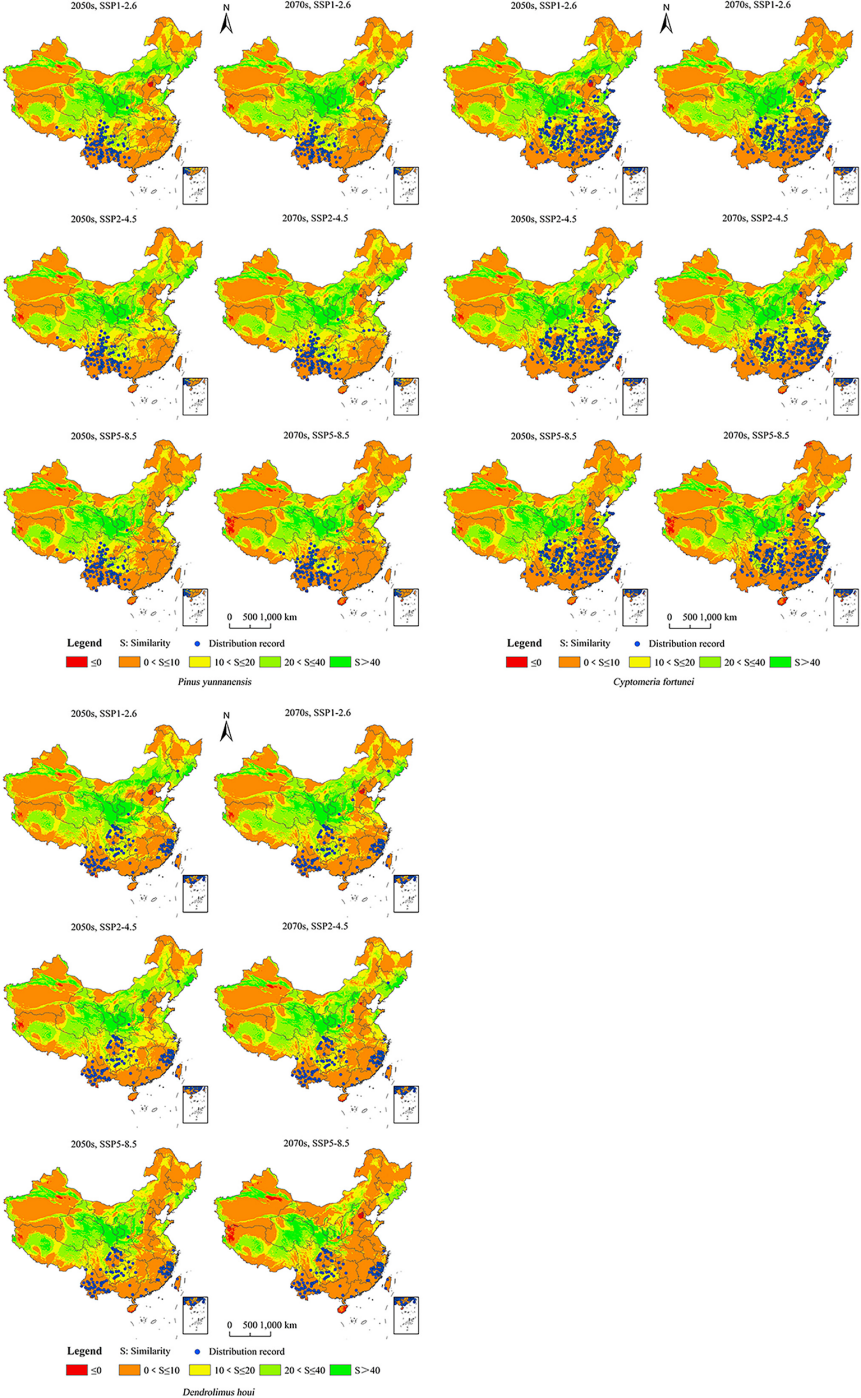
MESS maps for possible distribution areas of *P. yunnanensis*, *C. fortunei*, and *D. houi* under scenarios of future climate change.

All three future climate scenarios showed small potential distribution areas of the climate anomaly (S ≤ 0, red area in **Figure 7**). Comparing the possible area of distribution under the same scenario of climate change for the same future period (**Figure 7**; **Table S5**), the area suitable for the growth of *P. yunnanensis* did not occur in the climate anomaly area. Under the climate scenarios of the 2050s under SSP1–2.6, 2050s under SSP2–4.5, 2050s under SSP5–8.5, 2070s under SSP1–2.6, 2070s under SSP2–4.5, and 2070s under SSP5–8.5, the 112 points representing the current distribution of *P. yunnanensis* were 7.81, 8.47, 8.31, 8.98, 8.09, and 6.94, respectively. This result indicated that the anomaly under SSP5–8.5 in the 2070s exceeded those of the remaining two climate scenarios. The 238 points representing the current distribution of *C. fortunei* were 7.91, 6.95, 8.90, 8.45, 6.33, and 5.58, respectively, representing a higher anomaly in the 2070s under SSP5–8.5 compared to that in the remaining two climate scenarios. The 111 points representing the current distribution of *D. houi* were 6.36, 7.08, 7.15, 5.99, 6.03, and 4.01, respectively, representing a higher anomaly for the 2070s under SSP5–8.5 compared to that under the remaining climate scenarios.

## Discussion

### Role of climatic variables in restricting the potential geographical distribution

Optimizing MaxEnt with Enmeval data package can reduce both fitting degree and sampling deviation, thus improving prediction accuracy (Sony et al., 2018). After optimization, the training data of AUC values of the three species were all higher than 0.9, indicating that the prediction accuracy was very high. The present study used the MaxEnt model to simulate the environmental characteristics of *D. yunnanensis* and its host plants, so as to evaluate the likely distribution area of *D. houi* and study the main environment variables affecting this pest. The consistency of the validation statistics for the host-plant and pest predictions demonstrated the robustness and reliability of the MaxEnt model in predicting areas highly suitable for pest development. The main variables affecting the potential distribution area of *D. houi* and its host plants were isothermality (bio3), temperature seasonality (bio4), maximum temperature of warmest month (bio5), minimum temperature of warmest month (bio6), and average temperature of coldest quarter (bio11). This indicates that the habitats suitable for *D. houi* and its host plants were mainly affected by temperature, which is closely related to the life history of *D. houi.* In March, the overwintering eggs of *D. houi* begin to hatch and feed on the tender leaves of shrubs. After the larvae have molted once or twice, the larvae are slightly larger and begin to feed on pine needles. From May to June, they change from young to older, mature larvae. From September to October, the adults emerge. In mid- and late November, the eggs begin to overwinter in the pine forest, the larvae hatching when the plant grows new leaves in the coming year (Yin et al., 2002). Zhao and Liu (2007) showed that extreme temperature and sunshine duration were significantly positively correlated with population fluctuation and occurrence area in *D. houi*. The higher the temperature, the better the development of *D. houi* worm eggs (Zhao and Liu, 2007). Climate change affects the distribution of invasive species *Leptocybe* invasa, among which temperature has the greatest influence on its potential distribution (Zhang et al., 2021). Climate is an important player in the outbreak of pine wood nematode disease, and the pine wood nematode disease frequently occurs in hot and dry conditions (Gruffudd et al., 2016). Temperature is the key factor affecting insect distribution area (Simberloff, 2000), and an increase in temperature will inevitably have an important impact on the geographical and vertical distribution of insects. An increase in temperature increases the activity of insects, such as the blood-sucking behavior of mosquitoes, which accelerates the spread of pathogenic bacteria among humans (Yi et al., 2014). Environmental variables are closely related to the geographical distribution of insects. Among many environmental variables affecting the distribution of species fitness, only a few key factors play a leading role. The temperature rise associated with global climate change is having a direct impact on the growth and development, metabolic rate, survival and reproduction, migration, and diffusion of insects (Du et al., 2007). Insects are typical ectothermic animals and are extremely affected by changes in ambient temperature (Ma et al., 2015). Tropical insects can escape warming sun exposure by finding shade, orienting away from the sun, or reducing solar radiation (Bonebrake et al., 2014). In addition to directly affecting the growth and development of insects and their survival and reproduction, rising temperatures also affect the growth and developmental progress of plants. Due to the different responses of insects and plants to rising temperature, the phenological synchronization of insects and plants changes, thus affecting the normal feeding regimes and occurrence of pests (Pyke et al., 2016). The existence of host plants was the premise for studying the suitable distribution area of *D. houi* because host plants provide the necessary nutrients and habitat for the growth, development, survival, and reproduction of phytophagous pests (Braschler and Hill, 2007).

### Changes in the potential geographical distribution under future-climate scenarios

Under all future-climate scenarios studied, the predicted total suitable area for the three species in the 2050s and 2070s will increase to different degrees compared with the current period, except for *P. yunnanensis* in the 2070s under SSP5–8.5. The host species of *D. houi* are related to its distribution. For example, *P. yunnanensis* is mainly fed upon in southwest China, while *C. fortunei* is mainly fed upon in east China. The pest develops mainly on conifers at high altitudes, and it can occur in one to two generations per year, depending on the local climate (Han et al., 2019). Climate warming will contribute to the reproduction and survival of *Lymantria dispar* (Lepidoptera); under simulated future climate change, its colonization rate on poplar increased from 33% in 1991 to 100% in 2071 (Logan et al., 2007). Climate warming under SSP5–8.5, SSP2–4.5, and SSP1–2.6 for the 2030s and 2050s will expand the potential geographical distributions for the pest *Helicoverpa zea* in China (Zhao et al., 2022). Climate warming is conducive to the expansion of insects limited by low temperature to high latitudes and high altitudes. In these regions, the winters are shorter and the spring comes early, which makes the insects with limited distributions expand northwards at a speed of 6.1 km every 10 years (Raza et al., 2015). Climate warming may prompt species to spread into new temperature-friendly zones, while forcing them out of inhospitable ones (Hughes, 2000). Insects will gradually expand to higher altitudes and latitudes under climate warming (Hickling et al., 2006). At the same time, it may reduce the suitable range at low altitudes and low latitudes (Wilson et al., 2005; Thomas et al., 2006). Under different future-climate scenarios, the centroid position of the highly suitable habitat of *D. houi* is similar to the direction of movement of *P. yunnanensis*, and moves to the northeast as a whole. A similar distribution range and centroid movement direction indicate that *P. yunnanensis* will face greater disease risk in the future. The distribution area of the pest is mainly affected by climate change, interspecies interactions—such as with host trees—diffusion capacity, population state richness, natural enemies, and human activities. We must strengthen the detection of pest occurrences, track the migration and spread of insects in real time, and observe the transformation of insect physiology and ecology, all of which will play a key role in controlling the pest outbreaks. It is necessary to set up monitoring points in areas of their frequent occurrence and organize professional personnel to conduct regular inspections so as to find and deal with them as soon as possible in order to reduce the hazard. Trunk injection technique can be used as an effective method for the pest outbreak control. The pests are closely related to the anthropogenic factors such as trade, traffic and human activities (Ficetola et al., 2007; Bertelsmeier et al., 2017). In the future, the species distribution model incorporating factors related to human activities such as human footprint besides bioclimatic variables will be established.

## Conclusions

Based on actual distribution data and current (1970–2000) and future (2050s and 2070s) climatic data for *D. houi* and its hosts, *P. yunnanensis* and *C. fortunei*, a MaxEnt model based on optimized parameters was used to predict the potential distribution areas of the three species. The results indicated that the model fit was excellent and that the most significant climate variable affecting the potential suitable habitats was temperature. *Dendrolimus houi*, *P. yunnanensis*, and *C. fortunei* will move to areas of a higher altitude and higher latitude due to a warming climate. The migration directions of *P. yunnanensis* and *D. houi* were similar. The highly suitable and totally suitable habitats of all three taxa will expand under scenarios of future climate change (SSP2–4.5, SSP1–2.6, SSP5–8.5) in both the 2050s and 2070s, except that for *D. houi*, the highly suitable and totally suitable habitat will be lower under SSP5–8.5 in the 2070s. This study predicts the occurrence of and damage by *D. houi*, and provides a theoretical reference for the control of this pest.

## Data availability statement

The raw data supporting the conclusions of this article will be made available by the authors, without undue reservation.

## Author contributions

XO: conception and design of the research. XO and SB: acquisition of data. XO: analysis and interpretation of data. XO: statistical analysis. XO: drafting the manuscript and revising the manuscript. AC: acquire the fund. HL, JC and AC: managing the project and revising the manuscript too. All authors contributed to the article and approved the submitted version.

## Funding

This project was funded by the Key Research and Development Program of Zhejiang Province (No. 2019C02024)

## Conflict of interest

The authors declare that the research was conducted in the absence of any commercial or financial relationships that could be construed as a potential conflict of interest.

## Publisher’s note

All claims expressed in this article are solely those of the authors and do not necessarily represent those of their affiliated organizations, or those of the publisher, the editors and the reviewers. Any product that may be evaluated in this article, or claim that may be made by its manufacturer, is not guaranteed or endorsed by the publisher.
