# Supplementary material for: Simulation the potential distribution of Dendrolimus houi and its hosts, Pinus yunnanensis and Cryptomeria fortunei, under climate change in China
Source: Front Plant Sci. 2022 Nov 14;13:1054710. doi: 10.3389/fpls.2022.1054710 (PMC9703064; doi:10.3389/fpls.2022.1054710)
Supplement: Supplementary file 1 [file DataSheet_1.docx]

**Table S1.** Description of bioclimatic variables.

| Variable | Description | Variable | Description |
| --- | --- | --- | --- |
| bio1 | Annual mean temperature | bio11 | Average of coldest quarter |
| bio2 | Mean diurnal range (mean of monthly (max temp-min temp)) | bio12 | Annual precipitation |
| bio3 | Isothermality (bio2/bio7) (×100) | bio13 | Precipitation of wettest month |
| bio4 | Temperature seasonality (standard deviation×100) | bio14 | Precipitation of driest month |
| bio5 | maximum temperature of warmest month | bio15 | Precipitation seasonality (coefficient of variation) |
| bio6 | Minimum temperature of warmest month | bio16 | Precipitation of wettest quarter |
| bio7 | Annual mean temperature range | bio17 | Precipitation of driest quarter |
| bio8 | Mean temperature of wettest quarter | bio18 | Precipitation of warmest quarter |
| bio9 | Mean temperature of driest quarter | bio19 | Precipitation of coldest quarter |
| bio10 | Mean temperature of warmest quarter |  |  |

**Table S2.** Suitable areas at future risk of forest disease.

| Decade, scenario | *Pinus yunnanensis* predicted areas/104 km^2^ | | | |
| --- | --- | --- | --- | --- |
|  | Poorly suitable habitat | Moderately suitable habitat | Highly suitable habitat | Totally suitable habitat |
| Current | 93.53 | 32.51 | 34.55 | 160.59 |
| 2050s, SSP1–2.6 | 128.15 | 55.14 | 48.46 | 231.75 |
| 2050s, SSP2–4.5 | 153.39 | 55.26 | 48.66 | 257.31 |
| 2050s, SSP5–8.5 | 141.74 | 55.39 | 49.03 | 246.16 |
| 2070s, SSP1–2.6 | 128.42 | 49.36 | 44.36 | 222.14 |
| 2070s, SSP2–4.5 | 131.54 | 49.41 | 43.93 | 224.88 |
| 2070s, SSP5–8.5 | 98.06 | 28.21 | 29.67 | 155.94 |
| Decade, scenario | *Cryptomeria fortunei* predicted areas/104 km^2^ | | | |
|  | Poorly suitable habitat | Moderately suitable habitat | Highly suitable habitat | Totally suitable habitat |
| Current | 75.17 | 105.40 | 87.15 | 267.72 |
| 2050s, SSP1–2.6 | 47.30 | 73.25 | 164.89 | 285.44 |
| 2050s, SSP2–4.5 | 57.81 | 57.56 | 188.37 | 303.74 |
| 2050s, SSP5–8.5 | 49.16 | 52.63 | 200.47 | 302.26 |
| 2070s, SSP1–2.6 | 39.42 | 52.46 | 187.00 | 278.88 |
| 2070s, SSP2–4.5 | 66.99 | 72.22 | 169.69 | 308.9 |
| 2070s, SSP5–8.5 | 72.94 | 64.49 | 167.66 | 305.09 |
| Decade, scenario | *Dendrolimus houi* predicted areas/104 km^2^ | | | |
|  | Poorly suitable habitat | Moderately suitable habitat | Highly suitable habitat | Totally suitable habitat |
| Current | 93.10 | 61.85 | 56.82 | 211.77 |
| 2050s, SSP1–2.6 | 87.28 | 71.00 | 110.39 | 268.67 |
| 2050s, SSP2–4.5 | 90.72 | 72.51 | 115.32 | 278.55 |
| 2050s, SSP5–8.5 | 86.48 | 79.85 | 118.41 | 284.74 |
| 2070s, SSP1–2.6 | 89.46 | 99.99 | 84.09 | 273.54 |
| 2070s, SSP2–4.5 | 72.06 | 84.75 | 132.82 | 289.63 |
| 2070s, SSP5–8.5 | 120.07 | 99.04 | 130.20 | 349.31 |

**Table S3.** Changes in suitable areas at future risk of forest disease.

| Decade, scenario | *Pinus yunnanensis* predicted areas/10^4^ km^2^ | | |
| --- | --- | --- | --- |
|  | Gain | Loss | Stable |
| 2050s, SSP1–2.6 | 71.07 | 0.70 | 160.55 |
| 2050s, SSP2–4.5 | 101.35 | 5.13 | 156.00 |
| 2050s, SSP5–8.5 | 88.25 | 3.22 | 157.92 |
| 2070s, SSP1–2.6 | 64.72 | 4.18 | 157.17 |
| 2070s, SSP2–4.5 | 69.05 | 5.25 | 155.83 |
| 2070s, SSP5–8.5 | 35.41 | 40.01 | 120.80 |
| Decade, scenario | *Cryptomeria fortune* Predicted areas/104 km^2^ | | |
|  | Gain | Loss | Stable |
| 2050s, SSP1–2.6 | 47.27 | 1.62 | 190.58 |
| 2050s, SSP2–4.5 | 56.17 | 2.64 | 189.52 |
| 2050s, SSP5–8.5 | 62.06 | 1.20 | 190.86 |
| 2070s, SSP1–2.6 | 47.40 | 0.41 | 191.81 |
| 2070s, SSP2–4.5 | 53.66 | 4.12 | 188.03 |
| 2070s, SSP5–8.5 | 49.30 | 9.38 | 182.72 |
| Decade, scenario | *Dendrolimus houi* Predicted areas/10^4^ km^2^ | | |
|  | Gain | Loss | Stable |
| 2050s, SSP1–2.6 | 57.47 | 0.27 | 210.88 |
| 2050s, SSP2–4.5 | 67.24 | 0.10 | 211.02 |
| 2050s, SSP5–8.5 | 73.40 | 0.04 | 211.07 |
| 2070s, SSP1–2.6 | 62.56 | 0.22 | 210.93 |
| 2070s, SSP2–4.5 | 78.53 | 0.00 | 211.11 |
| 2070s, SSP5–8.5 | 138.40 | 0.03 | 211.03 |

**Table S4.** Centroid coordinates of suitable *P. yunnanensis, C. fortunei,* and *D. houi* under different climate-change scenarios.

| Decade, scenario | *Pinus yunnanensis* | | *Cryptomeria fortunei* | | *Dendrolimus houi* | | |
| --- | --- | --- | --- | --- | --- | --- | --- |
|  | Longitude (°) | Latitude (°) | Longitude (°) | Latitude (°) | Longitude (°) | | Latitude (°) |
| Current | 106.71 | 27.45 | 112.02 | 28.39 | 109.61 | 27.56 | |
| 2050s, SSP1–2.6 | 107.56 | 28.59 | 111.94 | 29.51 | 110.80 | 28.96 | |
| 2050s, SSP2–4.5 | 107.92 | 29.39 | 111.86 | 29.66 | 110.91 | 29.28 | |
| 2050s, SSP5–8.5 | 107.47 | 28.91 | 111.86 | 29.66 | 110.99 | 29.43 | |
| 2070s, SSP1–2.6 | 107.78 | 28.73 | 111.95 | 29.35 | 110.88 | 29.10 | |
| 2070s, SSP2–4.5 | 109.74 | 29.93 | 112.08 | 29.81 | 111.01 | 29.57 | |
| 2070s, SSP5–8.5 | 103.67 | 28.66 | 112.38 | 29.95 | 110.33 | 31.14 | |

**Table S5.** MESS for the potential distribution of *P. yunnanensis, C. fortunei,* and *D. houi* under future climate-change scenarios.

| Decade, scenario | *Pinus yunnanensis* | *Cryptomeria fortunei* | *Dendrolimus houi* |
| --- | --- | --- | --- |
| 2050s, SSP1–2.6 | 7.81 | 7.91 | 6.36 |
| 2050s, SSP2–4.5 | 8.47 | 6.95 | 7.08 |
| 2050s, SSP5–8.5 | 8.31 | 8.90 | 7.15 |
| 2070s, SSP1–2.6 | 8.98 | 8.45 | 5.99 |
| 2070s, SSP2–4.5 | 8.09 | 6.33 | 6.03 |
| 2070s, SSP5–8.5 | 6.94 | 5.58 | 4.01 |
