# Supplementary material for: Simulation the potential distribution of Dendrolimus houi and its hosts, Pinus yunnanensis and Cryptomeria fortunei, under climate change in China
Source: Front Plant Sci. 2022 Nov 14;13:1054710. doi: 10.3389/fpls.2022.1054710 (PMC9703064; doi:10.3389/fpls.2022.1054710)
Supplement: Supplementary file 2 [file DataSheet_2.docx]

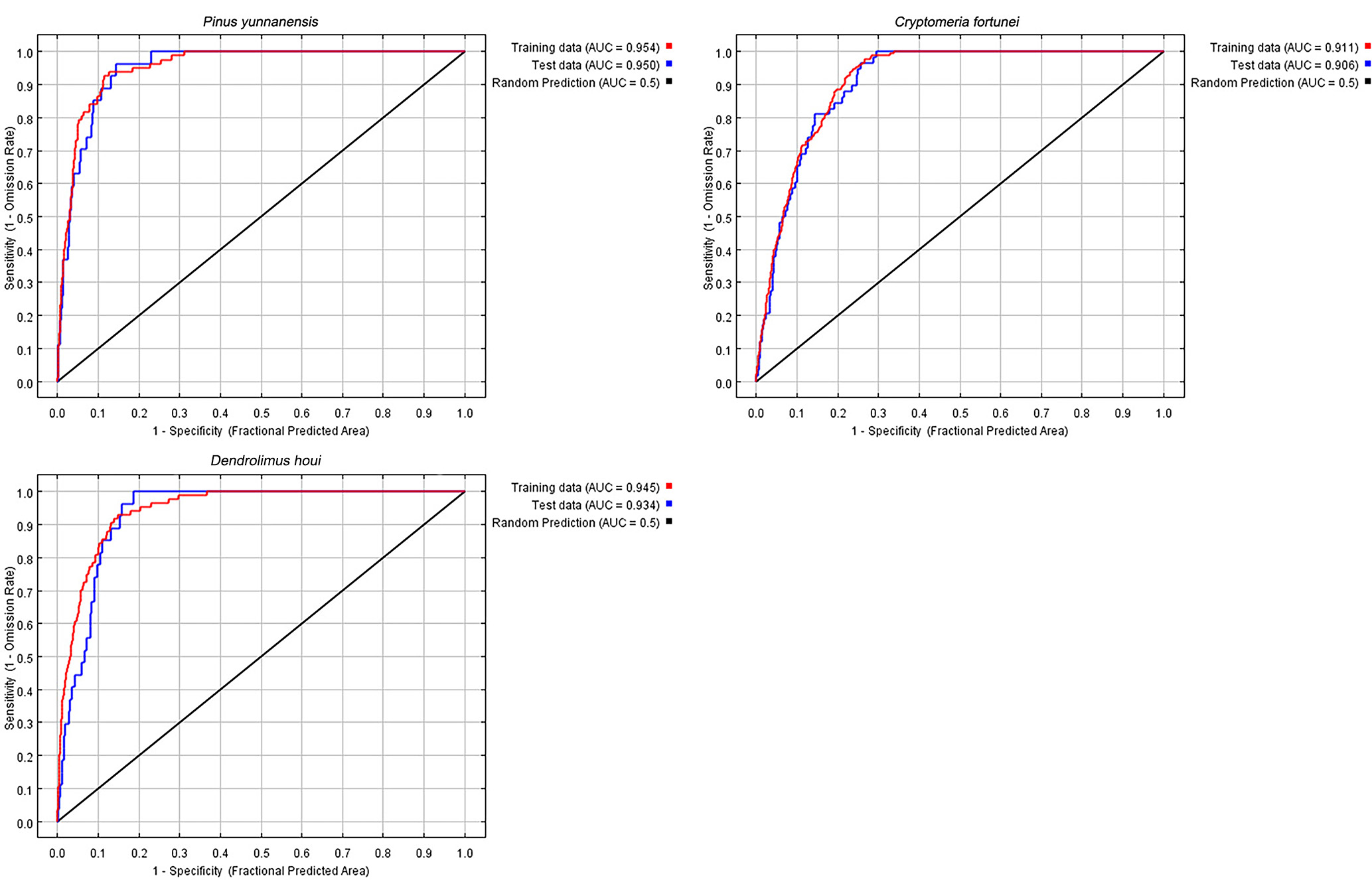


**Figure S1.** Assessment of reliability test of the distribution model created for *P. yunnanensis*, *C. fortunei*, and *D. houi*.
